# An integrated analysis of safety and tolerability of etelcalcetide in patients receiving hemodialysis with secondary hyperparathyroidism

**DOI:** 10.1371/journal.pone.0213774

**Published:** 2019-03-15

**Authors:** Geoffrey A. Block, Glenn M. Chertow, John T. Sullivan, Hongjie Deng, Omar Mather, Holly Tomlin, Michael Serenko

**Affiliations:** 1 Denver Nephrology, Denver, Colorado, United States of America; 2 Stanford University, Stanford, California, United States of America; 3 Amgen Inc., Thousand Oaks, California, United States of America; University of Milan, ITALY

## Abstract

**Background:**

Calcimimetics have been shown to be effective and safe therapies for the treatment of secondary hyperparathyroidism (sHPT), a serious complication of disordered mineral metabolism associated with dialysis-dependent chronic kidney disease. Etelcalcetide, a recently approved intravenous calcimimetic, reduces serum parathyroid hormone (PTH), calcium, phosphorus, and fibroblast growth factor-23 concentrations. Here we report the first integrated safety profile of etelcalcetide using pooled data from five pivotal clinical trials.

**Methods:**

This analysis included data from patients receiving hemodialysis with moderate to severe sHPT enrolled in two randomized, placebo-controlled trials; a randomized active-controlled (with cinacalcet) trial; and two single-arm, open-label extension trials. Patients initially received etelcalcetide intravenously 5 mg three times weekly (TIW) after hemodialysis; with potential dose increases of 2.5 or 5 mg at 4-week intervals to a maximum dose of 15 mg TIW, depending on serum PTH and calcium levels. The nature, frequency, and severity of treatment-emergent adverse events (AEs) and changes in laboratory parameters were assessed.

**Results:**

Overall, we evaluated 1023 patients from the placebo-controlled trials, 683 from the active-controlled trial, and 1299 from open-label extensions. The frequency and nature of common treatment-emergent AEs reported for the etelcalcetide arm were consistent among the placebo-controlled and active-controlled trials. The most common AEs were those related to mineral metabolism (decreased blood calcium, hypophosphatemia, muscle spasms) or gastrointestinal abnormalities (diarrhea, nausea, vomiting). Hypocalcemia leading to discontinuation of either calcimimetic was experienced in ≤ 1% of patients.

**Conclusions:**

This integrated safety assessment of etelcalcetide across placebo- and active-controlled trials showed an overall favorable risk/benefit profile, with safety similar to that of cinacalcet. Consistent with its mechanism of action, the most important risks associated with etelcalcetide were serum calcium reductions and hypocalcemia-related AEs; no new safety findings were identified in the pooled long-term extension trials.

## Introduction

Calcimimetics are effective and safe therapies for the treatment of secondary hyperparathyroidism (sHPT), a serious complication of disordered mineral metabolism affecting approximately 80% to 90% of patients receiving maintenance dialysis [1–4]. Secondary hyperparathyroidism begins as an adaptive response to maintain serum calcium and phosphorus homeostasis and is characterized by elevated serum concentrations of parathyroid hormone (PTH) mediated in part via the calcium sensing receptor (CaSR) on the parathyroid gland [1, 4]. If left untreated, sHPT often becomes refractory to therapy, which can contribute to demineralization of bone; ectopic vascular and visceral calcification; bone, joint, and muscle pain; pruritus; irritability; and hypertension, and has been associated with heightened risk of cardiovascular disease, hospitalization, and death [2, 5].

Etelcalcetide (Parsabiv, Amgen Inc., Thousand Oaks, CA), a recently approved calcimimetic, is an intravenously (IV) administered, long-acting peptide [6] that reduces serum PTH, calcium, phosphorus, and fibroblast growth factor-23 concentrations [7–10] via a mechanism of action similar to that of the oral calcimimetic cinacalcet (Sensipar/Mimpara, Amgen Inc., Thousand Oaks, CA). Calcimimetics, allosteric modulators of the CaSR, induce a conformational change in the receptor, increase sensitivity to extracellular calcium, and decrease circulating levels of PTH [4]. Although subtotal parathyroidectomy continues to be a treatment option, calcimimetic therapy has been shown to decrease the need for surgery [11, 12]. The recent update to the Kidney Disease Improving Global Outcomes (KDIGO) Clinical Practice Guideline suggests the use of calcimimetics, along with calcitriol or vitamin D analogs, as first-line options to lower PTH in patients receiving maintenance dialysis [13].

Among the most important risks associated with etelcalcetide are reductions in serum calcium and associated adverse events (AEs) that include neuromuscular irritability, seizures, QT prolongation, and ventricular arrhythmia [7, 9]. Herein we report the first integrated safety profile of etelcalcetide using pooled data from two randomized, placebo-controlled trials; a randomized active-controlled (with cinacalcet) trial; and two single-arm, open-label extension trials as part of the etelcalcetide development program (details on these trials, including ClinicalTrials.gov identifiers, are provided in the Patients and Trial Designs section).

## Materials and methods

### Patients and trial designs

**Fig 1:**
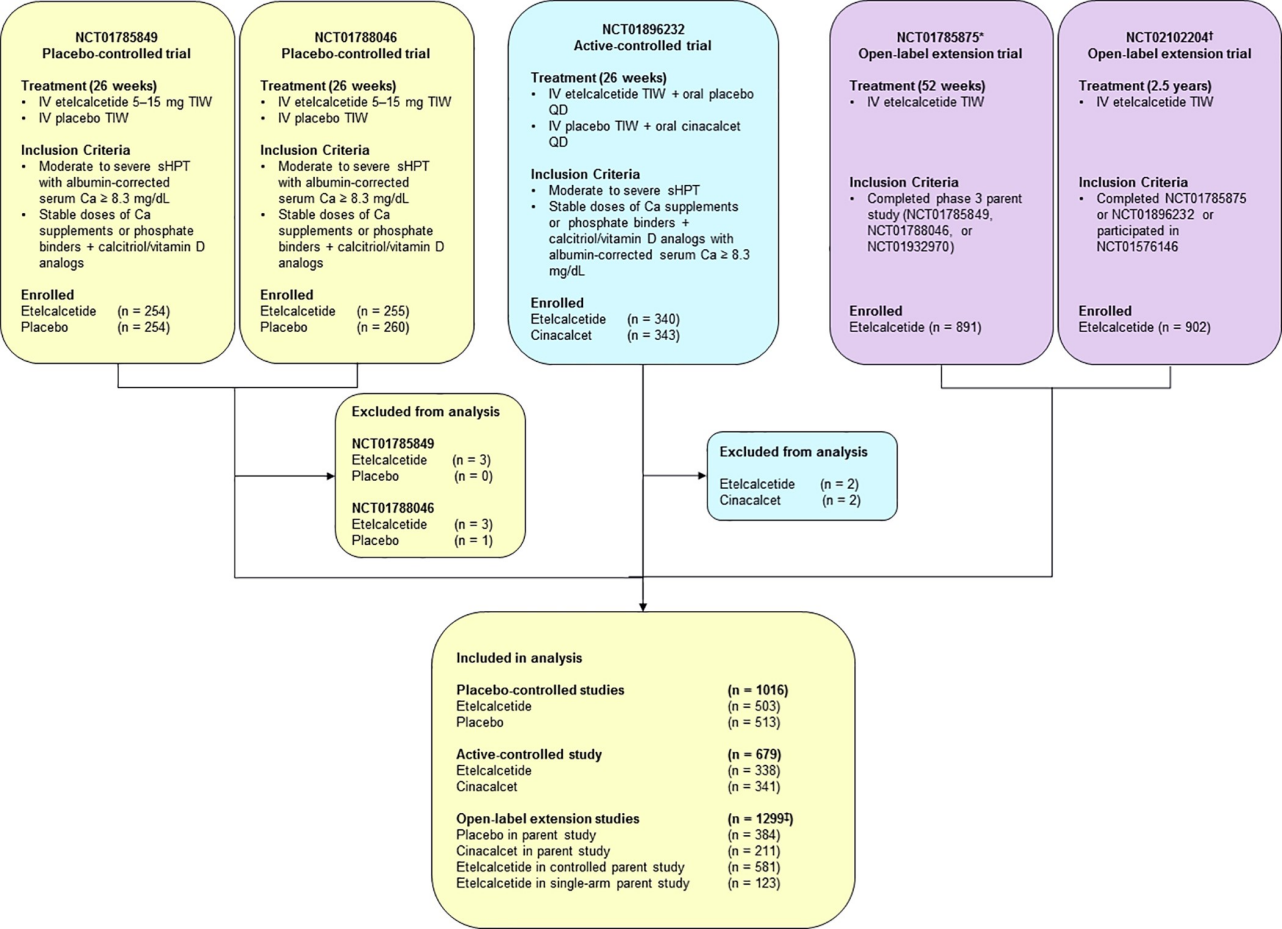
Trials included in the integrated safety analysis. *Trial NCT01785875 also contains data from patients enrolled from a single-arm parent trial (i.e., trial NCT01932970). ^†^Trial NCT02102204 also contains data from patients enrolled from a phase 2 parent trial (i.e., trial NCT01576146). ^‡^Indicates unique patients in the OLE pool. Ca = calcium; QD = every day; IV = intravenous; sHPT = secondary hyperparathyroidism; TIW = three times weekly.

Patients were on hemodialysis with moderate to severe sHPT (predialysis serum PTH > 400 pg/mL [placebo-controlled] or > 500 pg/mL [active-controlled]) and were receiving stable doses of calcium supplements, phosphate binders and/or calcitriol or active vitamin D analogs with an albumin-corrected serum calcium (cCa) ≥ 8.3 mg/dL. Data were from patients enrolled in five clinical trials: two 26-week, randomized, placebo-controlled trials (ClinicalTrials.gov identifiers: NCT01785849, NCT01788046) [9]; one 26-week, randomized, active-controlled trial (NCT01896232) [8]; and two single-arm, open-label extension trials (NCT01785875, NCT02102204). Descriptive information for each of these trials, including sample size, treatment duration, and inclusion criteria, is listed in **Fig 1**.

### Treatment

The starting dose of etelcalcetide was 5 mg IV three times weekly (TIW) given at the end of the hemodialysis session. Depending on serum PTH and calcium, the dose could be increased by 2.5 or 5 mg at 4-week intervals to a maximum dose of 15 mg TIW. Titration of etelcalcetide in the placebo-controlled and active-controlled trials was based on achieving a PTH concentration target of 100 to 300 pg/mL.

### Assessments

Patients were included in the safety analysis if they had received ≥ 1 dose of etelcalcetide. Safety was assessed by the nature, frequency, and severity of treatment-emergent AEs and by changes in laboratory parameters. Adverse events were coded using the Medical Dictionary for Regulatory Activities (MedDRA) version 17.1 and graded as mild, moderate, severe, or maximal/life-threatening (as assessed by the investigator). Treatment-emergent AEs were defined as AEs that started on or after the date of the first dose of investigational product and up to 30 days after the last dose of investigational product. Events of interest included blood calcium decreased (asymptomatic reductions in calcium < 7.5 mg/dL or clinically significant asymptomatic reductions in corrected serum calcium between 7.5 and 8.3 mg/dL), hypocalcemia (symptomatic reductions in corrected serum calcium < 8.3 mg/dL), hypophosphatemia, adynamic bone disease, fractures, cardiac failure, hypersensitivity reactions, infusion type reactions, effects on cardiac repolarization (i.e., QT prolongation and ventricular tachyarrhythmias), and upper gastrointestinal bleeding events [14]. Occurrences were summarized using specific groupings that were predefined using standardized MedDRA queries (SMQs) or Amgen Medical Queries criteria. Mean changes over time, shift tables, and outlier analyses were used in assessing blood chemistry and hematology parameters. The data were presented only for those parameters for which clinically significant abnormalities were noted.

### Statistical analyses

Descriptive statistics were presented for all safety analyses. The safety analysis set included all patients who had received ≥ 1 dose of etelcalcetide, placebo, or cinacalcet, depending on the individual trial. Crude percentage rates in the placebo-/active-controlled studies as well as the open-label extension studies were calculated using the number of patients experiencing an AE divided by the number of patients who were randomized to a particular treatment group. The exposure-adjusted incidence rate in the data set of pooled open-label extension studies was defined as the number of patients with a particular AE divided by the total exposure time among patients in the treatment group at risk of an initial occurrence of the event. Where applicable, the exposure-adjusted rates of selected AEs in the pooled open-label extension studies were compared with the placebo rates in the EVOLVE study [15], a large cardiovascular outcomes trial conducted in patients with chronic kidney disease receiving hemodialysis, that was used as an external control.

## Results

### Patients

In total, 1023 patients from the placebo-controlled trials (etelcalcetide, n = 509; placebo, n = 514), 683 patients from the active-controlled trial (etelcalcetide, n = 340; cinacalcet, n = 343), and 1299 patients from the open-label extension trials were included in this analysis (patients in the placebo- and active-controlled trials were eligible for the open-label extension trial; **Fig 1**). Overall, baseline demographics and characteristics were largely similar among the trials (**Table 1**). Baseline serum PTH concentrations were higher in patients in the active-controlled trial than in the placebo-controlled trials. The mean (range) duration of exposure for patients in the open-label extension was 554.3 (1–1063) days.

**Table 1:** Baseline characteristics and exposure.

	Placebo-Controlled Trials		Active-Controlled Trial		Open-Label Extension Trials
	Placebo (n = 514) n (%)	Etelcalcetide (n = 509) n (%)	Cinacalcet (n = 343) n (%)	Etelcalcetide (n = 340) n (%)	Etelcalcetide (n = 1299) n (%)
Mean (SD) age, year	58.1 (14.3)	58.4 (14.6)	55.3 (14.41)	54.0 (13.81)	56.8 (14.2)
Men, %	305 (59.3)	313 (61.5)	192 (56.0)	192 (56.5)	781 (60.1)
Race, %					
White	344 (66.9)	336 (66.0)	277 (80.8)	261 (76.8)	900 (69.3)
Black	149 (29.0)	136 (26.7)	52 (15.2)	54 (15.9)	329 (25.3)
Other	21 (4.1)	37 (7.3)	14 (4.1)	25 (7.4)	70 (5.4)
Median (Q1–Q3) time since initiation of dialysis, year	3.8 (1.8–6.9)	4.0 (2.0–7.8)	4.1 (1.7–7.5)	4.4 (2.0–7.8)	N/A
Median (Q1–Q3) PTH, pg/mL	716 (557–982)	724 (552–949)	930 (694–1327)	900 (685–1266)	N/A
Mean (SD) phosphate concentration, mg/dL	5.80 (1.53)	5.86 (1.59)	5.82 (1.58)	5.81 (1.69)	N/A
Mean (SD) calcium, albumin-corrected, mg/dL	9.65 (0.65)	9.64 (0.66)	9.58 (0.67)	9.67 (0.71)	N/A
Received ≥ 1 dose of investigational product	513	503	341	338	1298
Median (range) length of exposure, day	—	181.0 (1–190)	181.0 (3–197)	181.0 (1–190)	618.0 (1–1063)
Median (IQR) average weekly dose during EAP, mg/week	—	20.4 (8.9–30.0)	360.0 (185.0–562.9)	15.0 (9.2–30.0)	—

EAP = efficacy assessment phase; N/A = not available; PTH = parathyroid hormone.

### Safety

#### Overall

The frequency and nature of the common treatment-emergent AEs reported for the etelcalcetide arm of the placebo-controlled trials were consistent with those reported for the etelcalcetide arm of the active-controlled trial (**Table 2**). The most common AEs (≥ 10% of patients who received etelcalcetide) in the placebo-controlled trials were decreased blood calcium, muscle spasms, diarrhea, and nausea. Similarly, the most common AEs occurring in the etelcalcetide arm of the active-controlled trial were decreased blood calcium, nausea, and vomiting. No differences in hematologic parameters, liver enzymes or bilirubin, heart rate, or weight were observed between treatment groups. The most common AEs in the open-label extension trials were decreased blood calcium, diarrhea, vomiting, nausea, muscle spasm, and hypotension.

**Table 2:** Treatment-emergent adverse events occurring in ≥ 5% of patients in the etelcalcetide arms, adverse events leading to discontinuation, and serious adverse events.

	Placebo-Controlled Trials		Active-Controlled Trial		Open-Label Extension Trials
Preferred Term	Placebo (n = 513) n (%)	Etelcalcetide (n = 503) n (%)	Cinacalcet (n = 341) n (%)	Etelcalcetide (n = 338) n (%)	Etelcalcetide (n = 1298) n (%)
Number of patients with reported treatment-emergent AEs	410 (79.9)	461 (91.7)	307 (90.0)	314 (92.9)	1177 (90.7)
Blood calcium decreased	52 (10.1)	321 (63.8)	204 (59.8)	233 (68.9)	565 (43.5)
Diarrhea	44 (8.6)	54 (10.7)	35 (10.3)	21 (6.2)	168 (12.9)
Nausea	32 (6.2)	54 (10.7)	77 (22.6)	62 (18.3)	137 (10.6)
Vomiting	26 (5.1)	45 (8.9)	47 (13.8)	45 (13.3)	139 (10.7)
Muscle spasms	34 (6.6)	58 (11.5)	20 (5.9)	22 (6.5)	137 (10.6)
Hypotension	26 (5.1)	30 (6.0)	10 (2.9)	23 (6.8)	131 (10.1)
Hypertension	29 (5.7)	31 (6.2)	23 (6.7)	21 (6.2)	126 (9.7)
Hyperphosphatemia	9 (1.8)	9 (1.8)	10 (2.9)	10 (3.0)	113 (8.7)
Arteriovenous fistula site complication	26 (5.1)	29 (5.8)	6 (1.8)	9 (2.7)	104 (8.0)
Upper respiratory tract infection	26 (5.1)	21 (4.2)	15 (4.4)	10 (3.0)	104 (8.0)
Pain in extremity	20 (3.9)	24 (4.8)	14 (4.1)	17 (5.0)	102 (7.9)
Dyspnea	20 (3.9)	24 (4.8)	14 (4.1)	14 (4.1)	101 (7.8)
Back pain	19 (3.7)	22 (4.4)	10 (2.9)	9 (2.7)	97 (7.5)
Headache	31 (6.0)	38 (7.6)	24 (7.0)	22 (6.5)	93 (7.2)
Arthralgia	26 (5.1)	21 (4.2)	7 (2.1)	7 (2.1)	93 (7.2)
Hyperkalemia	11 (2.1)	22 (4.4)	15 (4.4)	13 (3.8)	93 (7.2)
Cough	22 (4.3)	22 (4.4)	9 (2.6)	10 (3.0)	94 (7.2)
Anemia	22 (4.3)	19 (3.8)	15 (4.4)	17 (5.0)	86 (6.6)
Abdominal pain	15 (2.9)	13 (2.6)	13 (3.8)	13 (3.8)	79 (6.1)
Viral upper respiratory tract infection	0	2 (0.4)	0	1 (0.3)	78 (6.0)
Pneumonia	17 (3.3)	11 (2.2)	3 (0.9)	6 (1.8)	77 (5.9)
Fall	14 (2.7)	15 (3.0)	8 (2.3)	3 (0.9)	76 (5.9)
Pyrexia	20 (3.9)	20 (4.0)	8 (2.3)	12 (3.6)	75 (5.8)
Fluid overload	11 (2.1)	16 (3.2)	7 (2.1)	7 (2.1)	66 (5.1)
Hypocalcemia	1 (0.2)	35 (7.0)	8 (2.3)	17 (5.0)	37 (2.9)
Patient incidence of treatment-emergent AEs leading to discontinuation of investigational product	13 (2.5)	9 (1.8)	16 (4.7)	19 (5.6)	39 (3.0)^a^
Number of patients reporting serious treatment-emergent AEs	149 (29.0)	130 (25.8)	93 (27.3)	85 (25.1)	671 (51.7)
Hyperkalemia	2 (0.4)	10 (2.0)	5 (1.5)	1 (0.3)	43 (3.5)
Pneumonia	14 (2.7)	10 (2.0)	1 (0.3)	1 (0.3)	56 (4.3)
Angina pectoris	3 (0.6)	7 (1.4)	1 (0.3)	1 (0.3)	15 (1.2)
Fluid overload	7 (1.4)	6 (1.2)	1 (0.3)	2 (0.6)	26 (2.0)
Atrial fibrillation	5 (1.0)	5 (1.0)	2 (0.6)	0	28 (2.2)
Cardiac failure congestive	5 (1.0)	5 (1.0)	1 (0.3)	0	24 (1.8)
Sepsis	4 (0.8)	4 (0.8)	4 (1.2)	3 (0.9)	42 (3.2)
Vascular graft thrombosis	5 (1.0)	3 (0.6)	3 (0.9)	0	15 (1.2)
Arteriovenous fistula thrombosis	5 (1.0)	2 (0.4)	1 (0.3)	1 (0.3)	33 (2.5)
Gangrene	2 (0.4)	2 (0.4)	0	4 (1.2)	9 (0.7)
Anemia	5 (1.0)	1 (0.2)	4 (1.2)	0	24 (1.8)

AE = adverse event.

^a^Based on N = 1289.

Adverse events leading to discontinuation of etelcalcetide were infrequent in the combined placebo-controlled trials and the active-controlled trial (**Table 2**). In the open-label extension trials, AEs leading to discontinuation of etelcalcetide included cardiac arrest, nausea, vomiting, hypocalcemia, general physical health deterioration, cellulitis, sepsis, and septic shock. In the placebo- and active-controlled trials, serious AEs occurred in approximately one-quarter of patients who received etelcalcetide; common serious AEs (≥ 2%) were hyperkalemia and pneumonia (**Table 2**). In the open-label extension trials, common serious AEs were pneumonia, hyperkalemia, sepsis, arteriovenous fistula thrombosis, cardiac arrest, atrial fibrillation, and fluid overload. The incidences of treatment-emergent AEs by time period are provided in the Supplement. No significant increases in the rates of AEs were noted within any of the system organ classes with prolonged exposure to etelcalcetide (**S1 Table**).

### Bone mineral metabolism

#### Effects on PTH, calcium, and phosphorus

Treatment with etelcalcetide effectively decreased PTH > 30% from baseline in the pooled placebo-controlled (etelcalcetide vs placebo) and active-controlled (etelcalcetide vs cinacalcet) trials: 74.7% versus 8.9% and 68.2% versus 57.7%, respectively [8, 9]. The PTH-lowering effects of etelcalcetide were sustained throughout the duration of the open-label trials. Decreased blood calcium was the most frequent AE across all trials and was mostly mild or moderate in severity. More patients in the etelcalcetide group experienced a mild decrease in blood calcium compared with the cinacalcet group (**Table 3**) in the active-controlled trial, with no difference noted in moderate or severe decreases in serum calcium. Importantly, no imbalance was noted in the incidence of serum cCa < 7.5 mg/dL between the etelcalcetide (26.5%) and cinacalcet (26.7%) groups in the active-controlled trial (**Table 3**). Rates of neuromuscular events potentially associated with low serum calcium were accordingly higher in the etelcalcetide groups compared with the placebo groups and mainly consisted of muscle spasms (11.5%, 6.6%), myalgia (1.6%, 0.2%), and paresthesia (4.8%, 0.6%)/hypoesthesia (1.8%, 0.8%), whereas the incidence of convulsions was low and similar between treatment groups (0.8%, 1.0%), respectively.

**Table 3:** Hypocalcemia in the placebo- and active-controlled trials.

	Placebo-Controlled Trials		Active-Controlled Trial	
	Placebo (n = 513) n (%)	Etelcalcetide (n = 503) n (%)	Cinacalcet (n = 341) n (%)	Etelcalcetide (n = 338) n (%)
Hypocalcemia, EOI	53 (10.3)	330 (65.6)	207 (60.7)	240 (71.0)
Hypocalcemia severity				
Mild^a^	38 (7.4)	213 (42.3)	124 (36.4)	177 (52.4)
Moderate^a^	14 (2.7)	141 (28.0)	84 (24.6)	70 (20.7)
Severe^a^	1 (0.2)	2 (0.4)	5 (1.5)	5 (1.5)
Life threatening	0	0	0	0
Unknown	0	0	0	0
Fatal	0	0	0	0
Leading to discontinuation of investigational product	0	5 (1.0)	2 (0.6)	0
Serious AEs	0	0	1 (0.3)	1 (0.3)
Blood calcium decreased	0	0	1 (0.3)	1 (0.3)
Number of patients with ≥ 1 postbaseline cCa value	511	499	337	336
cCa < 7.0 mg/dL	16 (3.1)	38 (7.6)	32 (9.5)	29 (8.6)
cCa < 7.5 mg/dL	28 (5.5)	135 (27.1)	90 (26.7)	89 (26.5)
cCa < 8.3 mg/dL	99 (19.4)	392 (78.6)	245 (72.7)	278 (82.7)

AE = adverse event; cCa = albumin-corrected serum calcium; EOI = event of interest.

^a^Mild, moderate, and severe categories as assessed by the investigator.

Hypophosphatemia was more common in the etelcalcetide groups compared with placebo or cinacalcet (**Table 4**); however, none of the hypophosphatemia events in the etelcalcetide arms were serious or resulted in discontinuation of drug.

**Table 4:** Patient incidence of treatment-emergent adverse events of interest.

	Placebo-Controlled Trials		Active-Controlled Trial		Open-Label Extension Trials
	Placebo (n = 513) n (%)	Etelcalcetide (n = 503) n (%)	Cinacalcet (n = 341) n (%)	Etelcalcetide (n = 338) n (%)	Etelcalcetide (n = 1298) n (%)
Adverse events of interest					
Hypocalcemia	53 (10.3)	330 (65.6)	207 (60.7)	240 (71.0)	586 (45.1)
Blood calcium decreased	52 (10.1)	321 (63.8)	204 (59.8)	233 (68.9)	565 (43.5)
Hypocalcemia	1 (0.2)	35 (7.0)	8 (2.3)	17 (5.0)	37 (2.9)
Adjusted calcium decreased	0	0	0	2 (0.6)	12 (0.9)
Chvostek’s sign	0	0	1 (0.3)	0	—
Hypophosphatemia events	2 (0.4)	7 (1.4)	3 (0.9)	5 (1.5)	32 (2.5)
Hypophosphatemia	1 (0.2)	7 (1.4)	3 (0.9)	2 (0.6)	26 (2.0)
Blood phosphorus decreased	1 (0.2)	0	0	3 (0.9)	6 (0.5)
Ventricular tachyarrhythmias	4 (0.8)	2 (0.4)	0	0	13 (1.0)
Ventricular tachycardia	0	2 (0.4)	0	0	2 (0.2)
Ventricular extrasystoles	2 (0.4)	0	0	0	3 (0.3)
Ventricular fibrillation	1 (0.2)	0	0	0	7 (0.5)
Ventricular tachyarrhythmia	1 (0.2)	0	0	0	0
Convulsions	5 (1.0)	4 (0.8)	2 (0.6)	3 (0.9)	21 (1.6)
Hypersensitivity	19 (3.7)	22 (4.4)	17 (5.0)	19 (5.6)	83 (6.4)
Infusion reaction^a^	29 (5.7)	29 (5.8)	16 (4.7)	23 (6.8)	110 (8.5)

AE = adverse event; cCa = albumin-corrected serum calcium; EOI = event of interest.

^a^Infusion reaction as listed in the Infusion Reaction EOI (narrow search) with onset day coinciding with investigational product infusion which were resolved on the same day or the day after onset.

#### Bone fractures

In the placebo-controlled trials, the patient incidence of clinical bone fractures was lower in the etelcalcetide group (1.6%) compared with the placebo group (2.9%) and similar between arms in the active-controlled trial (etelcalcetide, 2.1%; cinacalcet, 2.6%). Patients were not screened for asymptomatic fractures during any of the trials. The proportion of patients with sustained PTH < 100 pg/mL during the efficacy assessment phase (weeks 20–27 of the placebo- and active-controlled trials) was generally low for both the placebo-controlled trials (etelcalcetide, 7.0%; placebo, 0.4%) and the active-controlled trial (etelcalcetide, 6.7%; cinacalcet; 3.2%; **S2 Table**). The exposure-adjusted incidence for clinical bone fractures from the pooled open-label studies was 3.3 per 100 patient-years of exposure. This rate was consistent with the background rate of 4.9 per 100 patient-years of exposure in the placebo group of the EVOLVE trial (data on file), a large cardiovascular outcomes trial conducted in patients with chronic kidney disease receiving hemodialysis [15].

### Cardiac repolarization

A reduction in serum calcium was associated with QTc interval prolongation. Patients in the etelcalcetide groups experienced a maximum increase from baseline in the corrected QT interval using Fridericia’s formula (QTcF) of > 30 to 60 ms (19.7%) or > 60 ms (1.2%) more frequently compared with placebo (5.7%, 0%). Additionally, the patient incidence of maximum postbaseline predialysis QTcF > 480 to 500 ms in the etelcalcetide and placebo groups was 7.2% and 5.5%, respectively, and > 500 ms was 4.8% and 1.9%, respectively. QTc data were not collected in the active-controlled trial.

Ventricular arrhythmias occurred infrequently (< 1%), at a similar rate between the etelcalcetide (0.4%, n = 2) and placebo groups (0.8%, n = 4) and included ventricular tachycardia, ventricular extrasystole, ventricular fibrillation, and ventricular tachyarrhythmia (**Table 4**). None of these events were associated with hypocalcemia. No ventricular arrhythmias were reported in the active-controlled trial. In the pooled open-label extension trials, 13 patients (0.6 per 100 patient-years of exposure) experienced events of ventricular tachyarrhythmias.

Decreases in serum potassium were similar between groups (both < 1%). The incidence of increases to grade 3 hyperkalemia (> 6.0 to 7.0 mmol/L) was more frequent in patients who received etelcalcetide compared with placebo (6.4% vs 2.9%). Shifts to grade 4 hyperkalemia (> 7.0 mmol/L) were similar between treatment groups (etelcalcetide, < 1%; placebo, 1.0%).

### Heart failure

In the combined placebo-controlled trials, a numerical imbalance was noted in the rate of adjudicated congestive heart failure (CHF) events requiring hospitalization in the etelcalcetide treatment group (2.2%, n = 11) compared with the placebo group (1.2%, n = 6). Further analysis showed that the imbalance in the CHF events was limited to trial NCT01785849, where the rate of these events was 2.8% in the etelcalcetide group compared with 0.8% in the placebo group; no imbalances were noted in trial NCT01788046 (1.6% vs 1.5%, respectively). This observation could be attributed to a significant imbalance in baseline characteristics of trial NCT01785849 in the domain of ischemic heart disease. In this trial, more patients in the etelcalcetide group compared with placebo had a medical history of coronary artery disease (35.4% etelcalcetide; 29.9% placebo), myocardial infarction (15.7% etelcalcetide; 11.8% placebo), angina pectoris (13.0% etelcalcetide; 10.6% placebo), cerebrovascular accidents (11.0% etelcalcetide; 7.9% placebo), coronary artery bypass surgery (11.8% etelcalcetide; 5.5% placebo), and cardiac pacemaker insertion (4.3% etelcalcetide; 2.8% placebo). No such imbalances were noted in trial NCT01788046.

During the adjudication process, review of the medical records showed that the majority of patients in the etelcalcetide and placebo groups who developed heart failure during the trials had a history of heart failure. Heart failure events were not adjudicated in the active-controlled or open-label extension trials.

### Upper gastrointestinal bleeding

In the placebo-controlled trials, the patient incidence of gastrointestinal bleeding was similar between the etelcalcetide and placebo groups (2.0% and 2.1%, respectively; **Table 5**). The rate in the etelcalcetide group (2.0%) was also similar to the rate in the placebo group (1.9%) of the EVOLVE trial. In clinical trials, two patients in the etelcalcetide group (combined 1253 patient-years of exposure) and no patients in the control (placebo- or active-) groups (combined 384 patient-years of exposure) had upper gastrointestinal bleeding noted at the time of death [7]. The exact cause of gastrointestinal bleeding in these patients was unknown, and there were too few cases to determine whether these cases were related to etelcalcetide.

**Table 5:** Patient incidence of treatment-emergent gastrointestinal bleeding events with historic control (EVOLVE).

	Placebo-Controlled Trials		Active-Controlled Trial		EVOLVE (First 6 Months)^a^	
	Placebo (n = 513) n (%)	Etelcalcetide (n = 503) n (%)	Cinacalcet (n = 341) n (%)	Etelcalcetide (n = 338) n (%)	Placebo (n = 1923) n (%)	Cinacalcet (n = 1938) n (%)
Gastrointestinal hemorrhage (SMQ)	11 (2.1)	10 (2.0)	5 (1.5)	9 (2.7)	36 (1.9)	45 (2.3)

EVOLVE = Evaluation of Cinacalcet Hydrochloride (HCl) Therapy to Lower Cardiovascular Events; SMQ = Standardized Medical Regulatory Dictionary for Regulatory Activities Query.

^a^Patient incidence rate using GI Hemorrhage SMQ during the first 6 months of the trial; data on file.

The exposure-adjusted rate of gastrointestinal bleeding events (3.1 per 100 patient-years) in the pooled open-label extension trials was similar to the rate of 3.9 per 100 patient-years observed in the placebo group of the EVOLVE trial that served as a historic control. Review of individual events revealed no common patterns of association with respect to dose-response, temporal association, or age.

### Immunogenicity

In completed clinical trials where immunogenicity of etelcalcetide has been evaluated, the incidence of antietelcalcetide antibodies (evaluated using a surface plasmon resonance−based immunoassay) was relatively low (71/995; 7.1%), and only 1.5% of patients (14/71) tested positive for the development of new antibodies [16]. The remaining 57 patients (5.7%) presented as antibody-positive before receiving etelcalcetide.

### Infusion and hypersensitivity reactions

In the placebo-controlled trials, incidences of events of interest in the infusion reactions category were similar between the etelcalcetide and placebo groups: 5.8% and 5.7%, respectively (**Table 4**). To increase the specificity of the search, only events with an onset day that coincided with etelcalcetide infusion and that resolved on the same day or on the day after the event onset were included in the analysis. With the exception of one event of syncope, which occurred and resolved several hours after discharge from the dialysis unit and was assessed as unrelated to etelcalcetide by the investigator, none of the possible infusion reactions were considered serious, and none resulted in discontinuation of etelcalcetide. In the pooled placebo-controlled trials, the rate of events in the hypersensitivity category was also similar between the etelcalcetide and placebo groups: 4.4% and 3.7%, respectively. No serious AEs indicative of anaphylactic-type reactions were identified. In the pooled open-label extension trials, infusion type reactions and hypersensitivity-related events were identified for 110 patients (5.6 per 100 patient-years of exposure) and 83 patients (4.2 per 100 patient-years of exposure), respectively. No confirmed events of infusion reaction or events indicative of anaphylaxis that could be attributed to etelcalcetide were reported during the open-label extension studies. One patient had anaphylactic reaction of moderate severity on trial day 181 and continued receiving etelcalcetide without interruption (the event was attributed to ceftriaxone).

## Discussion

This integrated assessment of the etelcalcetide safety profile did not reveal any safety signals beyond those already reported in the pivotal trials [8, 9]. Assessments were based on the pooled data from the two phase 3 placebo-controlled trials, the active-controlled trial, and two open-label extension trials with exposure up to 1063 days. Adverse drug reactions (i.e., events considered related to etelcalcetide) were mainly those secondary to decreased blood calcium (QT prolongation, parasthesia, muscle spasms, and myalgia) or related to gastrointestinal symptoms (diarrhea, nausea, and vomiting); other adverse drug reactions were hyperkalemia, heart failure, hypophosphatemia, and headache [7, 17, 18].

Hypocalcemia, an ontarget downstream effect of both etelcalcetide and cinacalcet, resulted in discontinuation of either calcimimetic in ≤ 1% of patients. The majority of cases of hypocalcemia observed after treatment with etelcalcetide were mild or moderate in severity; serious cases were infrequent: two serious events of hypocalcemia and two serious events of decreased blood calcium were noted across all etelcalcetide treatment arms (including long-term trials). No life-threatening or fatal cases of hypocalcemia occurred, and no difference in moderate or severe hypocalcemia rates were noted between patients treated with etelcalcetide or cinacalcet. Data from cell-based assays suggest an additive effect on CaSR signaling when etelcalcetide and cinacalcet are administered together. Therefore, etelcalcetide and cinacalcet should not be coadministered as this may lead to an increased risk of hypocalcemia [19]. Cinacalcet should be discontinued for at least 7 days before starting etelcalcetide, and corrected serum calcium must be at or above the lower limit of normal before etelcalcetide initiation [7]. The potential for hypocalcemia and its sequelae, as well as the potential for rapid rebound of calcium levels after an initial event of hypocalcemia [20], underscore the importance of frequent monitoring of blood calcium and other bone metabolism markers. The most recent KDIGO guidelines indicate that patients receiving treatment for sHPT specifically and chronic kidney disease/mineral and bone disorder more generally can reasonably be monitored more frequently for efficacy and adverse effects and that therapeutic decisions are to be based on trends rather than a single laboratory value, considering all available assessments of markers of bone metabolism markers [13].

Additional hypocalcemia-related AEs associated with cardiac depolarization, such as QTc prolongation and ventricular arrhythmias, occurred infrequently in patients who received etelcalcetide. There was no increased incidence in AEs potentially associated with QTc interval prolongation among patients receiving etelcalcetide compared with those receiving placebo. Ventricular arrhythmia events occurred at similar rates in patients who received etelcalcetide or placebo. There was no increase in the risk of ventricular arrhythmias observed with longer exposure to etelcalcetide in the open-label extension trials. The results of this analysis are consistent with preclinical evidence from canine trials suggesting that the QT prolongation associated with etelcalcetide results from decreases in serum calcium but not directly from etelcalcetide concentrations, and thus, there is no evidence of a direct effect of etelcalcetide on cardiac repolarization [21]. Moreover, no effects of etelcalcetide on human potassium ion channel Kv11.1 (ether-a-go-go or hERG) currents were observed in vitro [21] at concentrations approximately 30 times the maximum plasma-free drug concentrations achieved in patients in a clinical setting [22]. Although there is no evidence of a direct effect of etelcalcetide on cardiac repolarization, QT prolongation may occur secondary to drug-induced hypocalcemia. Serum corrected calcium should therefore be closely monitored.

Given the important role of PTH in maintaining bone remodeling, both pathologic elevations and reductions in PTH can result in an increased risk of skeletal fracture [2]. Despite the occurrence of PTH < 100 pg/mL (albeit at a low rate), this analysis showed a fracture rate in the etelcalcetide group that was almost half of that observed in the placebo group, and no increase in the rate of fractures was noted with the prolonged exposure to etelcalcetide. Moreover, calcimimetics have been shown to increase bone density through normalization of PTH. The BONAFIDE trial showed improvements in bone morphology associated with corresponding reductions in PTH, calcium, and markers of bone turnover [23]. Similar results were observed in a subanalysis of the EVOLVE trial [15]. Bone biopsy was not a protocol-specified procedure in the clinical trials; accordingly, the relationship between fracture events and possible oversuppression of PTH with a reduction in bone remodeling cannot be definitively determined. However, the lack of fractures in patients with PTH < 100 pg/mL, combined with decreases in the bone turnover markers bone-specific alkaline phosphatase and collagen type 1 cross-linked C-telopeptide to a greater extent in the etelcalcetide relative to the control groups [8, 9], suggest that overall etelcalcetide may result in positive effects on bone mass.

Chronic kidney disease is a strong and independent risk factor for cardiovascular disease, including heart failure; left ventricular hypertrophy and systolic dysfunction have been reported in approximately 73.9% and 14.8% of patients starting dialysis [24]. Although the rate of adjudicated heart failure hospitalizations was higher in patients in the etelcalcetide group (2.2%) compared with the placebo group (1.2%), this observation could be attributed to a significant imbalance in baseline characteristics of trial NCT01785849 in the domain of ischemic heart disease. In addition, the majority of patients with adjudicated heart failure events in both groups in the placebo-controlled trials had a history of heart failure.

Together with nonclinical toxicology trials that indicate no direct effect of etelcalcetide on myocardial function that could lead to new-onset or worsen preexisting heart failure [21], these data suggest that treatment with etelcalcetide is unlikely to have any adverse effect on the rates of new-onset heart failure. It is possible that an etelcalcetide-mediated reduction in calcium levels may trigger hemodynamic instability and that rapid or prolonged hypocalcemia may potentially reduce myocardial contractility, particularly in patients with impaired heart function; however, the role of low serum calcium in the development of CHF has not been established.

Upper gastrointestinal bleeding at the time of death was noted in two patients who received etelcalcetide compared with none who received placebo or cinacalcet. A review of the individual cases revealed both cases had multiple confounding factors, including comorbid conditions and the use of anticoagulant agents. Thus, although an association cannot be ruled out, the data do not suggest a causal link between etelcalcetide administration and upper gastrointestinal bleeding.

Events in the infusion reactions category were noted with equal frequency in patients treated with etelcalcetide or placebo and were generally mild to moderate, infrequent, and typically associated with symptoms or signs that commonly occur in this population, such as hypertension, hypotension, pyrexia, syncope, and wheezing [16]. Although no association was observed between exposure to etelcalcetide and the occurrence of infusion/hypersensitivity-type reactions in clinical trials, serious allergic reactions may potentially occur in the postmarketing setting.

Antibody impact analyses demonstrated that the presence of antietelcalcetide antibodies had no effect on the pharmacokinetics, efficacy, or safety of etelcalcetide [16]. Etelcalcetide is neither an inhibitor nor inducer of the hepatic cytochrome P450 (CYP450) enzymes (unlike cinacalcet, which is metabolized by CYP3A4 and CYP1A2 and inhibits CYP2D6); therefore, the risk of metabolic or pharmacokinetic drug-drug interactions with etelcalcetide is low to negligible [7, 22, 25, 26]. On the other hand, pharmacodynamic interactions may occur with other therapeutics that reduce serum calcium, and accordingly, caution should be exercised should this be needed. Etelcalcetide and cinacalcet should not be coadministered, given the added risk of hypocalcemia that results from the similar mechanisms of action and effects on serum levels of PTH, calcium, and phosphorous.

## Conclusion

In this integrated safety analysis, etelcalcetide exhibited a safety profile similar to that of cinacalcet; however, because of the limited experience with etelcalcetide in clinical practice, uncertainty remains regarding its safety profile in the real-world setting. Fortunately, an abundance of safety data exists for cinacalcet, which has a similar mechanism of action to etelcalcetide, and the many similarities in safety between these two calcimimetics should provide assurance for clinicians. Overall, these results indicate that risks associated with etelcalcetide use should be manageable with careful dose titration, periodic monitoring of serum calcium, and vigilance for AEs that may be prompted by hypocalcemia.

## Supporting information

S1 Table Incidence of treatment-emergent adverse events by system organ class and time period in the open-label extension trials. (DOCX)

S2 Table Patients with mean PTH < 100 pg/mL during the efficacy assessment phase. (DOCX)
