# Supplementary material for: An integrated analysis of safety and tolerability of etelcalcetide in patients receiving hemodialysis with secondary hyperparathyroidism
Source: PLoS One. 2019 Mar 15;14(3):e0213774. doi: 10.1371/journal.pone.0213774 (PMC6420005; doi:10.1371/journal.pone.0213774)
Supplement: S1 Table — (DOCX) [file pone.0213774.s001.docx]

# An integrated analysis of safety and tolerability of etelcalcetide in patients receiving hemodialysis with secondary hyperparathyroidism

Geoffrey A. Block^1^, Glenn M. Chertow^2^, John T. Sullivan^3^, Hongjie Deng^3^, Omar Mather^3^*, Holly Tomlin^3^, Michael Serenko^3^

^1^Denver Nephrology, Denver, Colorado, United States of America

^2^Stanford University, Stanford, California, United States of America

^3^Amgen Inc., Thousand Oaks, California, United States of America

*Corresponding author

E-mail: [omather@amgen.com](mailto:mserenko@amgen.com)

# Supporting information

## S1 Table. Incidence of Treatment-Emergent Adverse Events by System Organ Class and Time Period in the Open-Label Extension Trials

|  | **Weeks**  **1–26**  **n=1298**  **n (%)** | **Weeks 26–52**  **n=1288**  **n (%)** | **Weeks**  **52–78**  **n=1161**  **n (%)** | **Weeks 78–104**  **n=998**  **n (%)** | **Weeks 104–130**  **n=714**  **n (%)** | **Week 130+**  **n=545**  **n (%)** | **Total**  **N=1298**  **n (%)** |
| --- | --- | --- | --- | --- | --- | --- | --- |
| Investigations | 29 (2.2) | 432 (33.5) | 275 (23.7) | 164 (16.4) | 98 (13.7) | 56 (10.3) | 662 (51.0) |
| Infections and infestations | 24 (1.8) | 257 (20.0) | 238 (20.5) | 180 (18.0) | 137 (19.2) | 101 (18.5) | 564 (43.5) |
| Gastrointestinal disorders | 24 (1.8) | 243 (18.9) | 212 (18.3) | 125 (12.5) | 95 (13.3) | 54 (9.9) | 500 (38.5) |
| Injury, poisoning, and procedural complications | 25 (1.9) | 244 (18.9) | 203 (17.5) | 141 (14.1) | 110 (15.4) | 65 (11.9) | 490 (37.8) |
| Musculoskeletal and connective tissue disorders | 20 (1.5) | 215 (16.7) | 166 (14.3) | 116 (11.6) | 82 (11.5) | 54 (9.9) | 444 (34.2) |
| Metabolism and nutrition disorders | 15 (1.2) | 183 (14.2) | 133 (11.5) | 100 (10.0) | 63 (8.8) | 46 (8.4) | 393 (30.3) |
| Vascular disorders | 9 (0.7) | 154 (12.0) | 128 (11.0) | 95 (9.5) | 61 (8.5) | 41 (7.5) | 359 (27.7) |
| General disorders and administration site conditions | 17 (1.3) | 157 (12.2) | 126 (10.9) | 75 (7.5) | 65 (9.1) | 44 (8.1) | 352 (27.1) |
| Nervous system disorders | 13 (1.0) | 144 (11.2) | 115 (9.9) | 81 (8.1) | 61 (8.5) | 32 (5.9) | 336 (25.9) |
| Respiratory, thoracic, and mediastinal disorders | 20 (1.5) | 107 (8.3) | 114 (9.8) | 83 (8.3) | 56 (7.8) | 51 (9.4) | 311 (24.0) |
| Cardiac disorders | 8 (0.6) | 103 (8.0) | 96 (8.3) | 63 (6.3) | 45 (6.3) | 37 (6.8) | 281 (21.6) |
| Skin and subcutaneous tissue disorders | 10 (0.8) | 72 (5.6) | 53 (4.6) | 42 (4.2) | 24 (3.4) | 13 (2.4) | 176 (13.6) |
| Psychiatric disorders | 6 (0.5) | 50 (3.9) | 34 (2.9) | 23 (2.3) | 16 (2.2) | 13 (2.4) | 120 (9.2) |
| Blood and lymphatic system disorders | 5 (0.4) | 45 (3.5) | 34 (2.9) | 26 (2.6) | 16 (2.2) | 15 (2.8) | 119 (9.2) |
| Eye disorders | 2 (0.2) | 32 (2.5) | 22 (1.9) | 13 (1.3) | 14 (2.0) | 9 (1.7) | 85 (6.5) |
| Renal and urinary disorders | 3 (0.2) | 15 (1.2) | 23 (2.0) | 15 (1.5) | 5 (0.7) | 11 (2.0) | 61 (4.7) |
| Hepatobiliary disorders | 0 | 11 (0.9) | 19 (1.6) | 16 (1.6) | 9 (1.3) | 6 (1.1) | 54 (4.2) |
| Neoplasms benign, malignant, and unspecified | 0 | 20 (1.6) | 14 (1.2) | 9 (0.9) | 7 (1.0) | 4 (0.7) | 50 (3.9) |
| Ear and labyrinth disorders | 1 (0.1) | 11 (0.9) | 15 (1.3) | 10 (1.0) | 7 (1.0) | 4 (0.7) | 41 (3.2) |
| Reproductive system and breast disorders | 2 (0.2) | 16 (1.2) | 8 (0.7) | 9 (0.9) | 4 (0.6) | 3 (0.6) | 34 (2.6) |
| Endocrine disorders | 1 (0.1) | 11 (0.9) | 5 (0.4) | 5 (0.5) | 1 (0.1) | 2 (0.4) | 25 (1.9) |
| Immune system disorders | 0 | 5 (0.4) | 10 (0.9) | 6 (0.6) | 2 (0.3) | 1 (0.2) | 23 (1.8) |
| Congenital, familial, and genetic disorders | 0 | 3 (0.2) | 3 (0.3) | 3 (0.3) | 1 (0.1) | 1 (0.2) | 11 (0.8) |
| Surgical and medical procedures | 0 | 5 (0.4) | 3 (0.3) | 1 (0.1) | 0 | 0 | 9 (0.7) |
