# Supplementary material for: An integrated analysis of safety and tolerability of etelcalcetide in patients receiving hemodialysis with secondary hyperparathyroidism
Source: PLoS One. 2019 Mar 15;14(3):e0213774. doi: 10.1371/journal.pone.0213774 (PMC6420005; doi:10.1371/journal.pone.0213774)
Supplement: S2 Table — (DOCX) [file pone.0213774.s002.docx]

# An integrated analysis of safety and tolerability of etelcalcetide in patients receiving hemodialysis with secondary hyperparathyroidism

Geoffrey A. Block^1^, Glenn M. Chertow^2^, John T. Sullivan^3^, Hongjie Deng^3^, Omar Mather^3^*, Holly Tomlin^3^, Michael Serenko^3^

^1^Denver Nephrology, Denver, Colorado, United States of America

^2^Stanford University, Stanford, California, United States of America

^3^Amgen Inc., Thousand Oaks, California, United States of America

*Corresponding author

E-mail: [omather@amgen.com](mailto:mserenko@amgen.com)

# Supporting information

## S2 Table. Patients With Mean PTH < 100 pg/mL During the Efficacy Assessment Phase

|  | **Placebo-Controlled Trials** | | **Active-Controlled Trial** | |  |
| --- | --- | --- | --- | --- | --- |
|  | **Placebo**  **(n=514)**  **n (%)** | **Etelcalcetide**  **(n=509)**  **n (%)** | **Cinacalcet**  **(n=343)**  **n (%)** | **Etelcalcetide**  **(n=340)**  **n (%)** | |
| Patients with ≥ 1 PTH assessment during EAP | 456 | 456 | 310 | 298 | |
| Patients with mean PTH < 100 pg/mL during EAP | 2 (0.4) | 32 (7.0) | 10 (3.2) | 20 (6.7) | |

EAP=efficacy assessment phase (i.e., weeks 20–27); PTH=parathyroid hormone.
